# Cardiovascular magnetic resonance reference values of mitral and tricuspid annular dimensions: the UK Biobank cohort

**DOI:** 10.1186/s12968-020-00688-y

**Published:** 2020-12-17

**Authors:** Fabrizio Ricci, Nay Aung, Sabina Gallina, Filip Zemrak, Kenneth Fung, Giandomenico Bisaccia, Jose Miguel Paiva, Mohammed Y. Khanji, Cesare Mantini, Stefano Palermi, Aaron M. Lee, Stefan K. Piechnik, Stefan Neubauer, Steffen E. Petersen

**Affiliations:** 1 Department of Neuroscience, Imaging and Clinical Sciences, “G.D’Annunzio” University, Chieti, Italy; 2 Department of Clinical Sciences, Lund University, Malmö, Sweden; 3 Casa Di Cura Villa Serena, 65013 Città Sant’Angelo, Pescara Italy; 4 William Harvey Research Institute, NIHR Barts Biomedical Research Centre, Queen Mary University of London, Charterhouse Square, London, EC1M 6BQ UK; 5 Barts Heart Centre, St Bartholomew’s Hospital, Barts Health NHS Trust, West Smithfield, London, UK; 6 Division of Cardiovascular Medicine, NIHR BRC Oxford, Radcliffe Department of Medicine, University of Oxford, Level 6, West Wing, John Radcliffe Hospital, Headington, Oxford, OX3 9DU UK

**Keywords:** Cardiovascular magnetic resonance, Reference values, Mitral valve, Tricuspid valve, Annulus, Tenting area, Tethering

## Abstract

**Background:**

Mitral valve (MV) and tricuspid valve (TV) apparatus geometry are essential to define mechanisms and etiologies of regurgitation and to inform surgical or transcatheter interventions. Given the increasing use of cardiovascular magnetic resonance (CMR) for the evaluation of valvular heart disease, we aimed to establish CMR-derived age- and sex-specific reference values for mitral annular (MA) and tricuspid annular (TA) dimensions and tethering indices derived from truly healthy Caucasian adults.

**Methods:**

5065 consecutive UK Biobank participants underwent CMR using cine balanced steady-state free precession imaging at 1.5 T. Participants with non-Caucasian ethnicity, prevalent cardiovascular disease and other conditions known to affect cardiac chamber size and function were excluded. Absolute and indexed reference ranges for MA and TA diameters and tethering indices were stratified by gender and age (45–54, 55–64, 65–74 years).

**Results:**

Overall, 721 (14.2%) truly healthy participants aged 45–74 years (54% women) formed the reference cohort. Absolute MA and TA diameters, MV tenting length and MV tenting area, were significantly larger in men. Mean ± standard deviation (SD) end-diastolic and end-systolic MA diameters in the 3-chamber view (anteroposterior diameter) were 2.9 ± 0.4 cm (1.5 ± 0.2 cm/m^2^) and 3.3 ± 0.4 cm (1.7 ± 0.2 cm/m^2^) in men, and 2.6 ± 0.4 cm (1.6 ± 0.2 cm/m^2^) and 3.0 ± 0.4 cm (1.8 ± 0.2 cm/m^2^) in women, respectively. Mean ± SD end-diastolic and end-systolic TA diameters in the 4-chamber view were 3.2 ± 0.5 cm (1.6 ± 0.3 cm/m^2^) and 3.2 ± 0.5 cm (1.7 ± 0.3 cm/m^2^) in men, and 2.9 ± 0.4 cm (1.7 ± 0.2 cm/m^2^) and 2.8 ± 0.4 cm (1.7 ± 0.3 cm/m^2^) in women, respectively. With advancing age, end-diastolic TA diameter became larger and posterior MV leaflet angle smaller in both sexes. Reproducibility of measurements was good to excellent with an inter-rater intraclass correlation coefficient (ICC) between 0.92 and 0.98 and an intra-rater ICC between 0.90 and 0.97.

**Conclusions:**

We described age- and sex-specific reference ranges of MA and TA dimensions and tethering indices in the largest validated healthy Caucasian population. Reference ranges presented in this study may help to improve the distinction between normal and pathological states, prompting the identification of subjects that may benefit from advanced cardiac imaging for annular sizing and planning of valvular interventions.

## Introduction

Multimodality cardiovascular imaging plays a major role in the diagnosis, prognosis, and management of valvular heart disease [1]. As a general rule, valvular regurgitant lesions may present a challenge for most diagnostic modalities because of their dynamic nature and dependency on hemodynamic and physiologic conditions [2]. An integrative approach is therefore recommended to achieve an accurate evaluation of the severity of the lesion and its clinical significance [3].

Transthoracic echocardiography is the first-line imaging modality for valvular heart disease assessment and provides the core of the evaluation of valvular regurgitation severity [4]. Cardiovascular magnetic resonance (CMR) provides complementary and highly accurate information about valve morphology, severity of the regurgitant lesion and cardiac remodelling, delivering unique insight into the mechanism of regurgitation and additional information for optimal timing of intervention [5, 6].

Quantitative assessment of mitral annulus (MA) and tricuspid annulus (TA) is essential in understanding mechanisms underlying valve regurgitation, and normative data, permitting the differentiation between normal and pathological states, are even more so to inform surgical planning. Recognizing abnormal valvular apparatus geometry and annular dilation from standard anatomical planes might help identify patients that may benefit from advanced cardiac imaging and interventions [7, 8]. However, reference ranges for MA and TA dimensions were hitherto available only for echocardiography [9, 10].

Based on the UK Biobank participant demographics and health status in ~ 5000 consecutive participants from the early phase of CMR [11, 12] acquisitions, we aim to select validated normal healthy Caucasian participants in order to establish balanced steady-state free precession (bSSFP)-based reference values for MA and TA dimensions from standard CMR two-dimensional long-axis views.

## Methods

### Study population

CMR examinations of 5065 consecutive UK Biobank participants were evaluated. Participants with non-Caucasian ethnicity, known cardiovascular disease, hypertension, respiratory disease, diabetes mellitus, hyperlipidemia, hematological disease, renal disease, rheumatological disease, malignancy, symptoms of chest pain or dyspnea, current- or ex-tobacco smokers, those taking medication for diabetes, hyperlipidemia or hypertension and those with body mass index (BMI) ≥ 30 kg/m^2^ were excluded from the analyses. We further excluded individuals with valvular heart disease of any type and severity. In order to create evenly distributed age-decade groups (45–54, 55–64, 65–74 years), all participants older than 74 years were also excluded from the cohort. Age- and sex-specific reference ranges for the left ventricle, right ventricle and atria from the same study population has been previously reported [13].

### CMR protocol

The full UK Biobank CMR protocol has been extensively described elsewhere [12]. Briefly, all CMR examinations were performed in Cheadle, United Kingdom, on a clinical wide bore 1.5 T scanner (MAGNETOM Aera, Syngo Platform VD13A, Siemens Healthineers, Erlangen, Germany). Assessment of MA and TA was performed based on the combination of long axis cines (4-chamber, 2-chamber and 3-chamber views) acquired at one slice per breath-hold. All acquisitions used bSSFP with typical parameters (subject to standard radiographer changes to planning), as follows: repetition time/echo time = 2.6/1.1 ms (2.7/1.16 ms for long axis), flip angle 80°, Grappa factor 2, voxel size 1.8 mm × 1.8 mm × 8 mm (6 mm for long axis). The actual temporal resolution of 32 ms was interpolated to 50 phases per cardiac cycle (~ 20 ms). No signal or image filtering was applied besides distortion correction.

### Image analysis

**Fig. 1:**
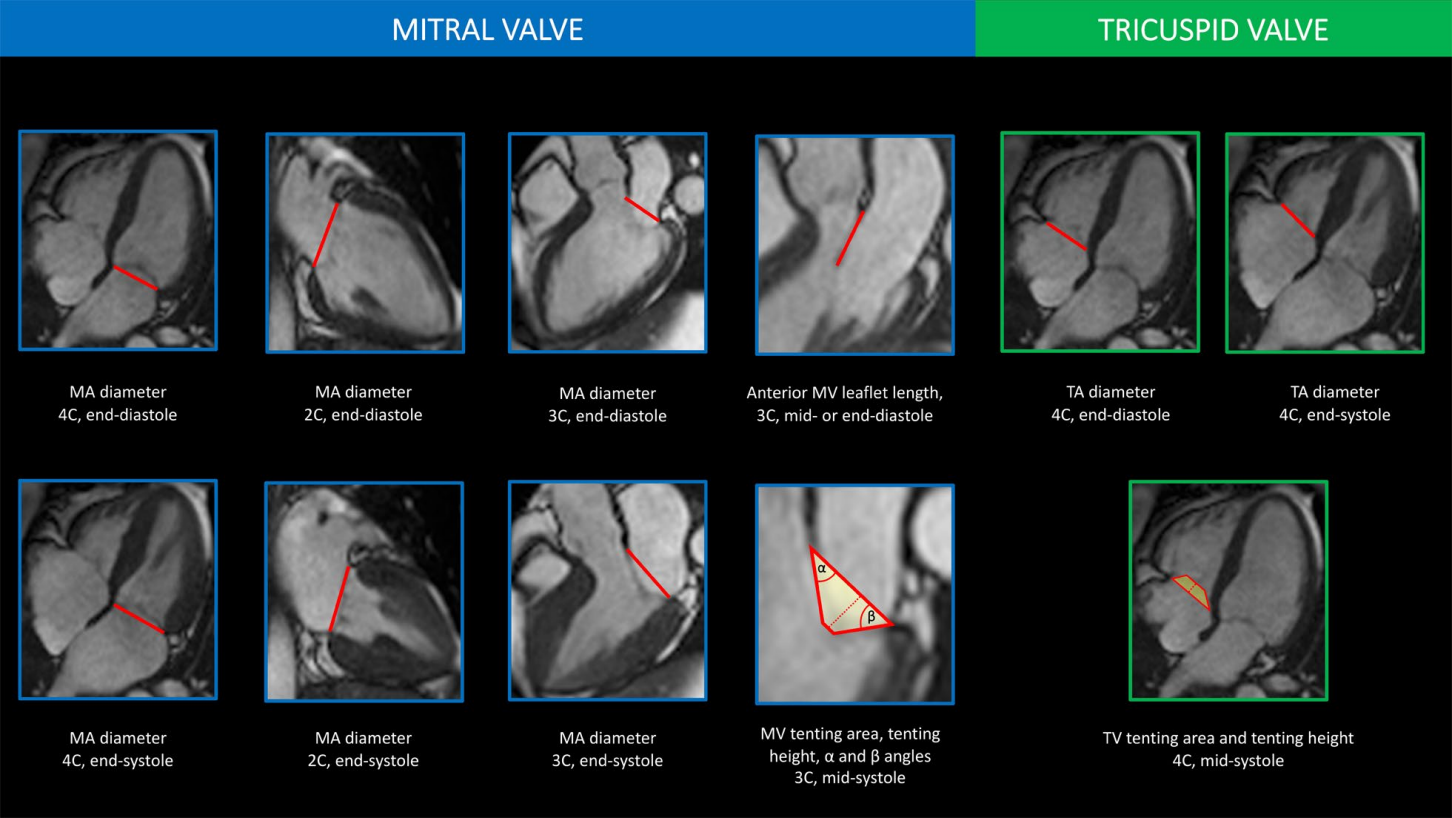
Mitral annular and tricuspid annular dimensions and tethering indices. 3C, 3-chamber view; 4C, 4-chamber view; MA, mitral annulus; MV, mitral valve; TA, tricuspid annulus; TV, tricuspid valve

Standard operating procedures for the analysis of MA and TA dimensions and tethering indices were developed and approved prior to study initiation (Fig. 1). CMR scans were analysed using CVi42 post-processing software (Version 5.1.1, Circle Cardiovascular Imaging Inc., Calgary, Canada).

### Mitral valve measurements

Two operators (F.R. and N.A.) performed linear measurement of MA on both end-systolic (frame with the largest left atrium) and end-diastolic (frame with the smallest left atrium) cardiac phases in horizontal long axis (HLA) (4-chamber) and vertical long axis (VLA) (2-chamber) and sagittal left ventricular outflow tract (LVOT) (3-chamber; conventional anteroposterior annulus) views. Mitral valve (MV) tenting area and tenting height (depth) were assessed in 3-chamber view on the mid-systolic cardiac phase (the frame midway between MV closure and end-systole) [4]. The length of the anterior mitral leaflet (AML) was measured in the 3-chamber view, from the most distal part of the leaflet up to its insertion during mid- or end-diastole, using the last diastolic image where the AML was most clearly visible. All measurements were then indexed to body surface area (BSA) or to height. Furthermore, we measured α and β angles that the mitral leaflets create with the MA line during mid-systole in the 3-chamber view, and the ratio of posterior to anterior leaflet tethering angle, representing a measure of the tethering pattern (the higher the ratio, the more asymmetric the pattern) [14]. Finally, we computed the ratio of diastolic antero-posterior MA diameter to AML length [15].

### Tricuspid valve measurements

Two operators (F.R. and N.A.) performed linear measurement of TA on both end-systolic (frame with the largest right atrium) and end-diastolic (frame with the smallest right atrium) cardiac phase in HLA (4-chamber) view. TV tenting area and tenting height (or depth) were assessed in 4-chamber view on the mid-systolic cardiac phase [4].

Additionally, we computed the ratio of end-diastolic TA to MA diameter in HLA (4-chamber) view.

Inter- and intra-observer variability between analysts for MA and TA measurements was assessed by analysis of fifty randomly selected CMR examinations, repeated after a one-month interval.

### Statistical analysis

All data are presented as mean ± standard deviation, unless stated otherwise. Continuous variables were visually assessed for normality using histograms and Q−Q plots. Independent sample Student’s t-test was used to compare mean values of CMR parameters between men and women. Outliers were defined a priori as CMR measurements more than three interquartile ranges below the first quartile or above the third quartile and removed from the analyses. Mean values for all cardiac parameters are reported by gender and age subgroups (45–54, 55–64, 65–74). An extension of the Wilcoxon rank-sum test was used to test the significance of trends across age subgroups [16]. Reference ranges for measured data are defined as the 95% prediction interval which is calculated by mean ± t_0.975, n-1_ (√(n + 1)/n) (SD) [17]. Absolute values of MA and TA dimensions and tethering indices were also indexed to BSA (using the DuBois and DuBois formula [18]) and height.

The reference ranges for the whole cohort were defined as the range where the measured value fell within the 95% prediction interval for the whole cohort regardless of age decade. The borderline zone was defined as the upper and lower ranges where the measured value lay outside the 95% prediction interval for at least one age group. The abnormal zone was defined as the upper and lower ranges, where the measured values were outside the 95% prediction interval for any age group [13]. Intra-class correlation coefficients (ICC) were obtained to assess inter- and intra-observer variability. Two-way ICC (2,1) was computed for inter-observer ICCs, to reflect the fact that a sample of cases and a sample of raters were observed, whereas a one-way ICC (1,1) was computed for intra-observer ICC [19]. A p-value < 0.05 was considered statistically significant for all tests. Statistical analysis was performed using R (version 3.3.0, Statistical Software, R Foundation for Statistical Computing, Vienna, Austria).

## Results

Of 5065 CMR scans acquired between 30th April 2014 and 30th August 2015, 91 examinations were discarded as either the CMR data were of insufficient quality or CMR identifier, and subject identifier did not match. Outliers (n = 10) and individuals with pair-wise missing data for annular measurements (n = 72) were also excluded. Finally, 721 (14.5%) subjects met the inclusion criteria. The baseline characteristics of the reference cohort are presented in Table 1. The mean age of the cohort was 59 ± 7 years (range 45–74) years. Men were taller, heavier, and had higher BSA values. A summary of absolute and indexed CMR parameters stratified by gender is presented in Additional file 1: Tables S1–S3 and further stratification by age and gender is reported in Additional file 1: Tables S4–S9.

**Table 1:** Baseline characteristics of the normal control study population

	Age subgroups (years)		
	45–54	55–64	65–74
Number of participants	224	302	195
Age (years)	51 (2)	59 (3)	68 (2)
Male gender	103 (46)	142 (47)	83 (43)
Systolic blood pressure (mmHg)	126 (14)	133 (16)	136 (18)
Diastolic blood pressure (mmHg)	76 (9)	77(9)	76 (9)
Heart rate (bpm)	66.9 (9.9)	68.4 (12.1)	68.6 (10.6)
Weight (kg)	70.6 (12.7)	69.9 (12.0)	67.7 (11.3)
Height (m)	1.72 (0.09)	1.70 (0.09)	1.69 (0.09)
BSA (m^2^)	1.83 (0.20)	1.81 (0.19)	1.77 (0.18)
BMI (kg/m^2^)	24.2 (2.9)	24.4 (2.7)	24.2 (2.8)

All continuous values are reported in mean ± standard deviation (SD), while categories are reported as number (percentage)

BMI, body mass index; BSA, body surface area

CMR reference ranges of MA and TA dimensions and tethering indices are provided in a traffic light format for males and females regardless of their age groups for both absolute and indexed values (Tables 2, 3, 4, 5, Additional file 1: Tables S10, S12). Age-specific reference ranges are also provided in tables dedicated to measured CMR values in the borderline (yellow) zone (Tables 6, 7, 8, 9, Additional file 1: Tables S11–S13).

**Table 2:**
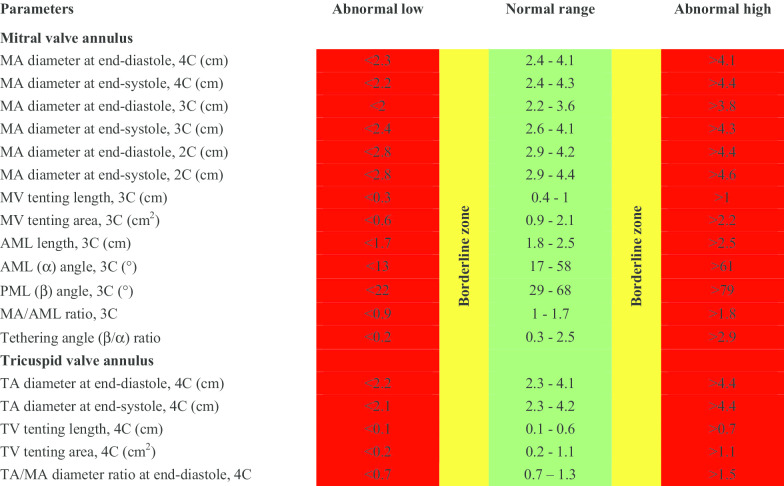
Absolute normal reference ranges of mitral and tricuspid valve annulus measurements in Caucasian men

Abnormal low and high refer to the lower and upper reference limits, respectively. They are defined as measurements which lie outside the 95% prediction interval at all age groups. Borderline zone values should be looked up in the age-specific tables. The borderline zone was defined as the upper and lower ranges where the measured value lay outside the 95% prediction interval for at least one age group. All measurements are reported in cm (areas in cm^2^), except for ratios and angles

2C, two-chamber; 3C, three-chamber; 4C, four-chamber; AML, anterior mitral leaflet; BSA, body surface area; MA, mitral annulus; MV, mitral valve; PML, posterior mitral leaflet; TA, tricuspid annulus; TV, tricuspid valve

**Table 3:**
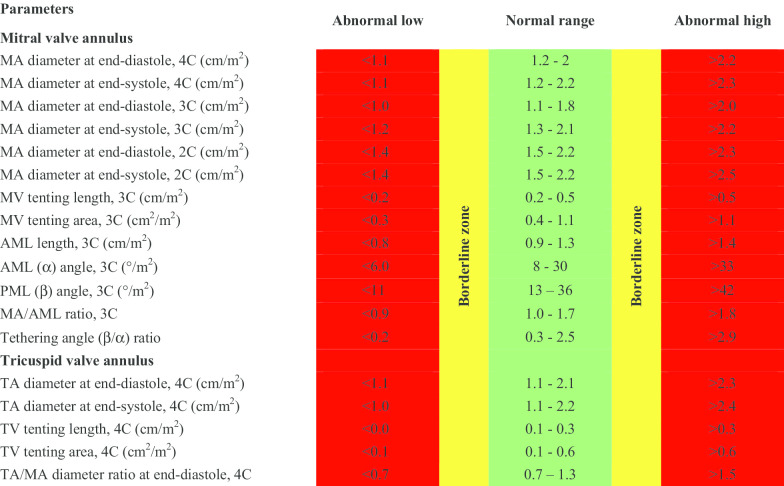
Normal reference ranges of mitral and tricuspid valve annulus measurements indexed to BSA in Caucasian men

Abnormal low and high refer to the lower and upper reference limits, respectively. They are defined as measurements which lie outside the 95% prediction interval at all age groups. Borderline zone values should be looked up in the age-specific tables. The borderline zone was defined as the upper and lower ranges where the measured value lay outside the 95% prediction interval for at least one age group. All measurements are indexed to body surface area and reported in cm/m^2^ (areas in cm^2^/m^2^), except for ratios and angles

2C, two-chamber; 3C, three-chamber; 4C, four-chamber; AML, anterior mitral leaflet; BSA, body surface area; MA, mitral annulus; MV, mitral valve; PML, posterior mitral leaflet; TA, tricuspid annulus; TV, tricuspid valve

**Table 4:**
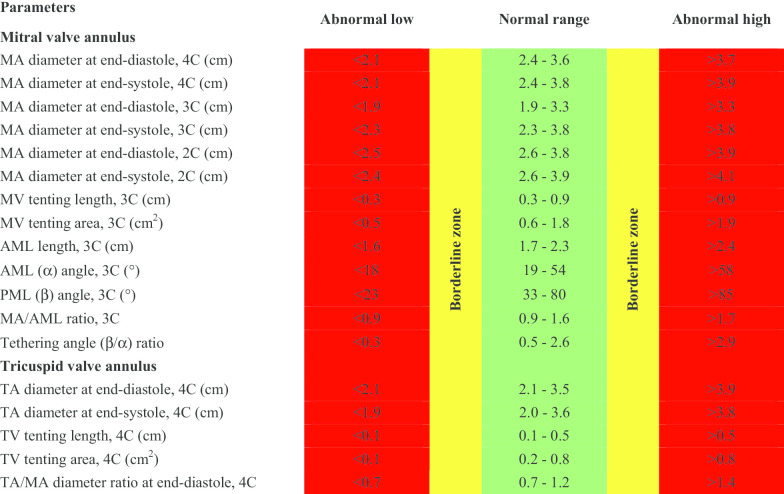
Normal reference ranges of mitral and tricuspid valve annulus measurements in Caucasian women

Abnormal low and high refer to the lower and upper reference limits, respectively. They are defined as measurements which lie outside the 95% prediction interval at all age groups. Borderline zone values should be looked up in the age-specific tables. The borderline zone was defined as the upper and lower ranges where the measured value lay outside the 95% prediction interval for at least one age group. All measurements are reported in cm (areas in cm^2^), except for ratios and angles

2C, two-chamber; 3C, three-chamber; 4C, four-chamber; AML, anterior mitral leaflet; BSA, body surface area; MA, mitral annulus; MV, mitral valve; PML, posterior mitral leaflet; TA, tricuspid annulus; TV, tricuspid valve.

**Table 5:**
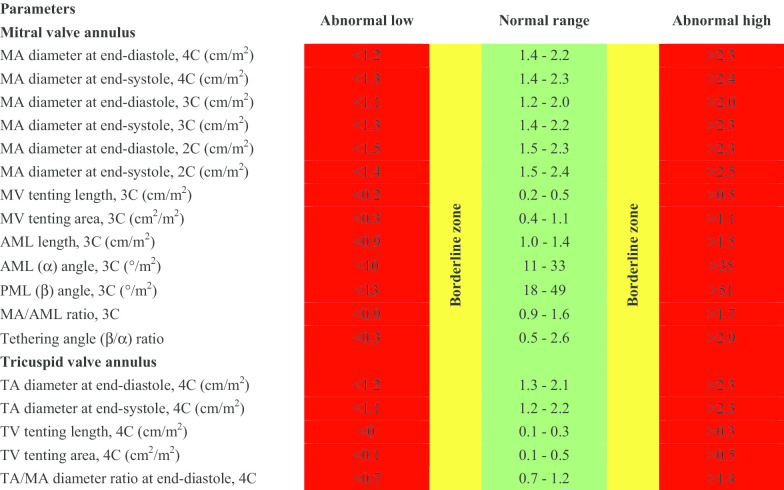
Normal reference ranges of mitral and tricuspid valve annulus measurements indexed to BSA in Caucasian women

Abnormal low and high refer to the lower and upper reference limits, respectively. They are defined as measurements which lie outside the 95% prediction interval at all age groups. Borderline zone values should be looked up in the age-specific tables. The borderline zone was defined as the upper and lower ranges where the measured value lay outside the 95% prediction interval for at least one age group. All measurements are indexed to body surface area and reported in cm/m^2^ (areas in cm^2^/m^2^), except for ratios and angles

2C, two-chamber; 3C, three-chamber; 4C, four-chamber; AML, anterior mitral leaflet; BSA, body surface area; MA, mitral annulus; MV, mitral valve; PML, posterior mitral leaflet; TA, tricuspid annulus; TV, tricuspid valve

**Table 6:**
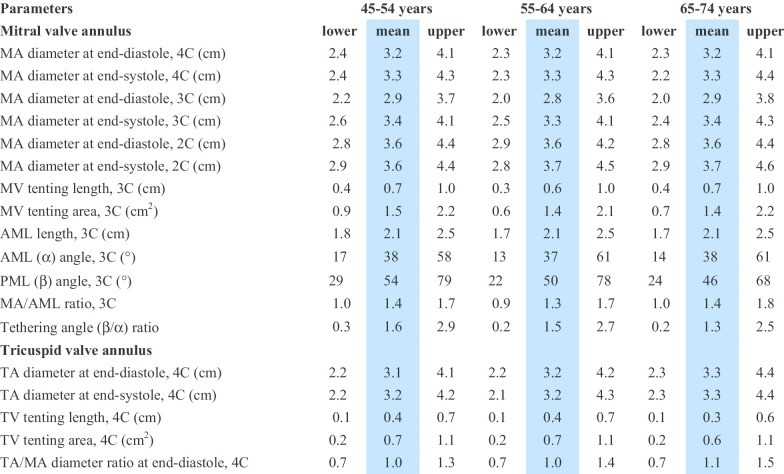
Age-specific absolute normal reference ranges of mitral and tricuspid valve annulus measurements in Caucasian men

Male reference ranges detailing mean, lower reference limit and upper reference limit by age group. Reference limits are derived by the upper and lower bounds of the 95% prediction interval for each parameter at each age group. All measurements are reported in cm (areas in cm^2^), except for ratios and angles

2C, two-chamber; 3C, three-chamber; 4C, four-chamber; AML, anterior mitral leaflet; BSA, body surface area; MA, mitral annulus; MV, mitral valve; PML, posterior mitral leaflet; TA, tricuspid annulus; TV, tricuspid valve

**Table 7:**
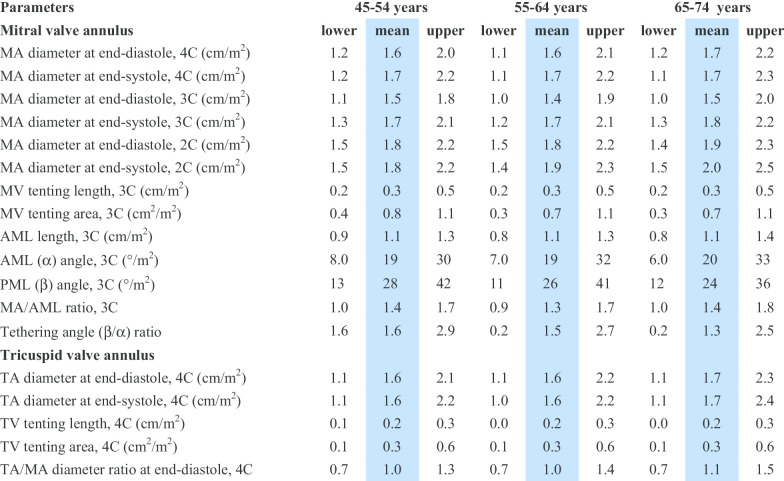
Age-specific normal reference ranges of mitral and tricuspid valve annulus measurements indexed to BSA in Caucasian men

Male reference ranges detailing mean, lower reference limit and upper reference limit by age group. Reference limits are derived by the upper and lower bounds of the 95% prediction interval for each parameter at each age group. All measurements are indexed to body surface area and reported in cm/m^2^ (areas in cm^2^/m^2^), except for ratios and angles

2C, two-chamber; 3C, three-chamber; 4C, four-chamber; AML, anterior mitral leaflet; BSA, body surface area; MA, mitral annulus; MV, mitral valve; PML, posterior mitral leaflet; TA, tricuspid annulus; TV, tricuspid valve

**Table 8:**
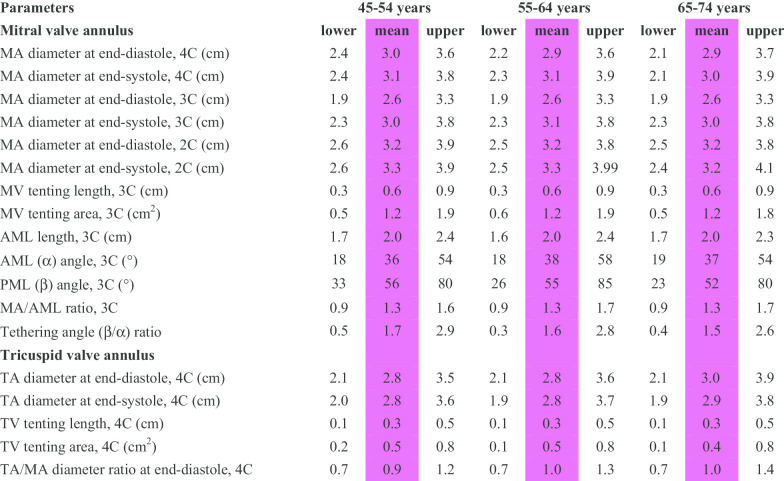
Age-specific absolute normal reference ranges of mitral and tricuspid valve annulus measurements in Caucasian women

Female reference ranges detailing mean, lower reference limit and upper reference limit by age group. Reference limits are derived by the upper and lower bounds of the 95% prediction interval for each parameter at each age group. All measurements are reported in cm (areas in cm^2^), except for ratios and angles

2C, two-chamber; 3C, three-chamber; 4C, four-chamber; AML, anterior mitral leaflet; BSA, body surface area; MA, mitral annulus; MV, mitral valve; PML, posterior mitral leaflet; TA, tricuspid annulus; TV, tricuspid valve

**Table 9:**
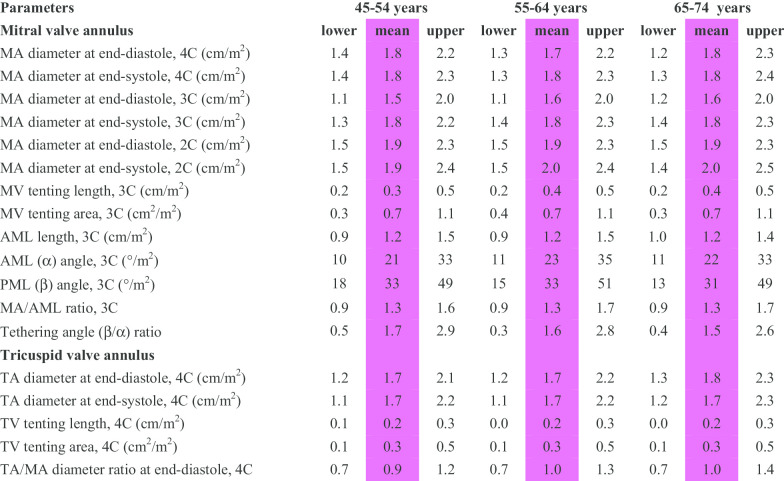
Age-specific normal reference ranges of mitral and tricuspid valve annulus measurements indexed to BSA in Caucasian women

Female reference ranges detailing mean, lower reference limit and upper reference limit by age group. Reference limits are derived by the upper and lower bounds of the 95% prediction interval for each parameter at each age group. All measurements are indexed to body surface area and reported in cm/m^2^ (areas in cm^2^/m^2^), except for ratios and angles

2C, two-chamber; 3C, three-chamber; 4C, four-chamber; AML, anterior mitral leaflet; BSA, body surface area; MA, mitral annulus; MV, mitral valve; PML, posterior mitral leaflet; TA, tricuspid annulus; TV, tricuspid valve

### Mitral valve measurements

Absolute MA diameters in 4C, 3C and 2C views, MV tenting length, MV tenting area, AML length and MA/AML ratio were significantly larger in men compared with women (Additional file 1: Table S1). However, after adjustment for BSA, women had larger MA diameters, AML length, AML and posterior mitral leaflet (PML) angles and tethering angle ratio (Additional file 1: Table S2). Conversely, after adjustment for height, MA diameters in 2C view, MV tenting area, MV tenting length and MA/AML ratio were larger in men, while AML and PML angles, and tethering angle ratio were larger in women (Additional file 1: Table S3).

With advancing age, both absolute and indexed MV tenting area trended down in men, while posterior MV leaflet angle and tethering angle ratio were smaller in both sexes (Additional file 1: Tables S4–S9).

### Tricuspid valve measurements

Absolute and height-indexed TA diameters, TV tenting length, TV tenting area and end-diastolic TA/MA diameter ratio were significantly larger in men (Additional file 1: Tables S1–S3, S8–S9). After adjustment for BSA, end-diastolic TA diameter was significantly larger in women (Additional file 1: Table S7).

With advancing age, TV tenting length trended down in men, while end-diastolic TA diameter and TA/MA diameter ratio became larger in both sexes (Additional file 1: Tables S4–S9).

### Intra- and inter-observer variability

Good to excellent intra- and inter-observer variability was achieved for all measurements with an inter-rater ICC between 0.92 and 0.98 and an intra-rater ICC between 0.90 and 0.97.

## Discussion

Over the last century, a sharp transition in the spectrum of valvular heart disease has been recognized. With longer lifespan expectancy most valve pathology is now degenerative, and the burden of valvular heart disease on healthcare systems is expected to increase. Mitral regurgitation is the most prevalent subtype of valvular heart disease in the general population, as well as the second most frequent indication for heart valve surgery [20]. Tricuspid regurgitation has been reported the second most common valvular heart disease after mitral regurgitation, namely in the elderly population, with a significant impact on survival [21]. Transcatheter valve technology has progressed and gained popularity as a suitable alternative for the treatment of valvular heart disease. This has renewed the interest in multimodality imaging of mitral and tricuspid valve, and mostly functional mitral regurgitation and tricuspid regurgitation, in which precise quantitative assessment of annular geometry is essential for understanding the mechanisms of regurgitation and planning of valvular intervention. However, quantitative measurements only provide meaningful information when compared to relevant reference values.

Our study provides absolute and indexed age- and gender-specific CMR reference ranges of MA and TA dimensions and indices of leaflet tethering based on the largest population-based dataset derived from a cohort of 721 Caucasian adults aged 45–74 rigorously free from pathophysiological or environmental risk factors affecting cardiac structure or function.

The measurements provided in this study identify normative data from standardized anatomical planes on two-dimensional bSSFP long-axis views, with somewhat expected differences from previously published echocardiographic thresholds.

Dilation of the MA is a key mechanism in the development and progression of both primary and secondary mitral regurgitation, whereas abnormal indices of leaflet tethering are major determinants of ischemic mitral regurgitation [2]. The cut-off for MA dilatation endorsed by the European Society of Cardiology is 3.5 cm (and/or MA/AML ratio > 1.3) [15], based on unindexed transoesophageal echocardiography measurement of the end-diastolic antero-posterior diameter of the MA performed in 49 anaesthetized patients scheduled for MV surgery [22]. However, subsequent echocardiographic studies reported wide inter-individual variability of MA measurements [23] and different thresholds between sexes [10]. In our study, mean ± SD end-diastolic and end-systolic MA diameters in the 3-chamber view (antero-posterior diameter) were 2.9 ± 0.4 cm (1.5 ± 0.2 cm/m^2^) and 3.3 ± 0.4 cm (1.7 ± 0.2 cm/m^2^) in men, and 2.6 ± 0.4 cm (1.6 ± 0.2 cm/m^2^) and 3 ± 0.4 cm (1.8 ± 0.2 cm/m^2^) in women, respectively. These results are consistent with previous evidence from Pini and colleagues who first observed gender differences in MV dimensions [24]. Particularly, the ratio of MV dimensions to BSA tended to be larger in normal women. Nevertheless, upon adjustment for height, antero-posterior MA dimensions were no longer different between sexes. Mean ± SD of MA/AML ratio was larger in men (1.4 ± 0.2) compared with women (1.3 ± 0.2). MA/AML ratio did not change significantly with age in either gender.

The most common methods proposed in the literature to quantify the degree of tethering include (i) tenting area, a simple area measurement from the leaflet tips to the annular plane performed at mid-systole, and (ii) coaptation height or depth, measuring the distance from the leaflet tips to the annular plane at mid-systole. Both parameters have been shown to correlate with the severity of secondary mitral regurgitation and mid-term postoperative outcome [25]. Importantly, annular dilation alone without leaflet tethering is an uncommon cause of significant secondary mitral regurgitation, such as in patients with left atrium dilatation from long-standing atrial fibrillation [26]. Furthermore, tethering angles define the geometrical relationship between the base of the leaflets to the annulus: α represents the angle between the annular plane and AML and β the angle between the annular plane and PML [27, 28]. Despite poor inter-modality agreement, a higher ratio of posterior to anterior angle—describing an asymmetric tenting phenotype—has been reported as a predictor of increased mitral regurgitation severity [14]. In our cohort, tethering angle ratio was larger in women (1.6 ± 0.6) compared with men (1.5 ± 0.6), and declining with ageing along with the reduction of β angle.

Accurate non-invasive measurement of TA size also has important clinical and surgical implications. The American College of Cardiology/American Heart Association (ACC/AHA) guidelines defines TA dilatation as an end-diastolic diameter > 40 mm or > 21 mm/m^2^ in the 4C transthoracic view and is the primary imaging criterion used to indicate severe tricuspid regurgitation [29]. However, these cut-offs were identified from apical 4-chamber echocardiographic view in studies not uniformly reporting the timing of the cardiac cycle to conduct TA measurement. Three-dimensional echocardiography captured higher thresholds for TA enlargement, reporting an upper limit of normal for TA diameter in the 4C view of 2.3 cm/m^2^[30], exactly matching our results in both sexes.

## Limitations

The normative reference ranges described were derived from a population of 45–74 years of Caucasian ethnicity, and may not be generalizable to other ethnic and age groups.

Measurements were performed exclusively from standardized anatomical planes on two-dimensional bSSFP long axis views routinely acquired as part of left ventricular structure and function module [31]; therefore inter-commissural annular diameter from modified 2C view was not available [32]. The complex anatomy of both MA and TA requires high-spatial resolution 3D imaging modalities for optimal evaluation of annular dimensions and geometry and should rely upon 3D whole-heart CMR data sets with multiplanar reformatting, as for 3D transesophageal echocardiography and cardiac computed tomography [33, 34]. Accordingly, in view of the expected suboptimal accuracy of the 2D-approach, we were also unable to adequately evaluate MA and TA area and short-axis dimensions. Although fully 3D dynamic reconstruction of MA and TA can be accurately performed by CMR, this is a time-consuming process typically practiced only in patients being considered for valve intervention [35]. Specific whole heart 3D CMR studies are needed to obtain reference values of TA and MA area and short-axis dimensions.

Our data were obtained from healthy subjects without valvular heart disease and therefore do not affect previous evidence that severe annular dilatation (> 5 cm in primary MR and > 3.7 cm in ischemic mitral regurgitation) is a predictor of unsuccessful repair [36]. This extends to the measurement of tethering indices, which have most frequently been evaluated in surgical series to assess the efficacy of MV and TV repair [25, 37].

CMR scans were not performed repeatedly on the same individuals over time; therefore, the associations described between age and CMR measurements are not longitudinal, but cross-sectional.

## Conclusions

This study provides age- and sex-specific normal reference ranges of MA and TA dimension and tethering indices from the largest healthy Caucasian population and will be useful in the analysis of CMR examinations in both clinical and research settings. Notably, reference ranges presented in our study may help to improve the distinction between normal and pathological states, prompting the identification of subjects that may benefit from advanced cardiac imaging for annular sizing and planning of valvular interventions.

## Supplementary information

**Additional file 1.** Additional reference ranges. Tables reporting additional normal reference ranges of mitral and tricuspid annular dimensions and tethering indices.

## Data Availability

This research has been conducted using the UK Biobank resource (see Appendix 1). The raw data, the derived data, the analysis and results will be clearly annotated and returned to UK Biobank, which will then incorporate the returned data into the central repository. UK Biobank will make the data available to all bona fide researchers for all types of health-related research that is in the public interest, without preferential or exclusive access for any person. All researchers will be subject to the same application process and approval criteria as specified by UK Biobank. Please see UK Biobank website for the detailed access procedure (https://www.ukbiobank.ac.uk/register-apply/).

## Appendix 1

### UK Biobank data

UK Biobank data in a codified tabular format, received through our access application (#2964), was used to select the healthy cohort. Data was translated, using the data dictionary provided as part of the application and the coding tables available through the UK Biobank website, into a self-contained table which we used to perform the analysis. The data derived from the analysis of CMR studies were tested for outlier values (defined as those values 3-times the interquartile range above the 3rd quartile or below the 1st quartile) that were removed from the final dataset.

## Abbreviations

ACC/AHA: American College of Cardiology/American Heart Assocation
AML: Anterior mitral leaflet
BMI: Body mass index
BSA: Body surface area
bSSFP: Balanced steady-state free precession
CMR: Cardiovascular magnetic resonance
HLA: Horizontal long axis
ICC: Intra-class correlation coefficient
LVOT: Left ventricular outflow tract
MA: Mitral annulus/mitral annular
MV: Mitral valve
PML: Posterior mitral leaflet
SD: Standard deviation
TA: Tricuspid annulus/tricuspid annular
TV: Tricuspid valve
VLA: Vertical long axis
