# Supplementary material for: Cardiovascular magnetic resonance reference values of mitral and tricuspid annular dimensions: the UK Biobank cohort
Source: J Cardiovasc Magn Reson. 2020 Dec 17;23:5. doi: 10.1186/s12968-020-00688-y (PMC7788733; doi:10.1186/s12968-020-00688-y)
Supplement: Supplementary file 1 — Additional file 1. Additional reference ranges. Tables reporting additional normal reference ranges of mitral and tricuspid annular dimensions and tethering indices. [file 12968_2020_688_MOESM1_ESM.docx]

**Table S1. Normal absolute reference ranges of mitral and tricuspid annulus measurements stratified by gender.**

| **Parameter** | **Male** | | **Female** | | | **p-value** |
| --- | --- | --- | --- | --- | --- | --- |
|  | **(n=328)** | | **(n=393)** | | |  |
|  | **Mean** | **SD** | **Mean** | **SD** | |  |
| **Mitral valve annulus** |  |  |  |  |  |  |
| MA diameter at end-diastole, 4C (cm) | 3.2 | 0.4 | 2.9 | 0.4 | <0.001 | |
| MA diameter at end-systole, 4C (cm) | 3.3 | 0.5 | 3.1 | 0.4 | <0.001 | |
| MA diameter at end-diastole, 3C (cm) | 2.9 | 0.4 | 2.6 | 0.4 | <0.001 | |
| MA diameter at end-systole, 3C (cm) | 3.3 | 0.4 | 3 | 0.4 | <0.001 | |
| MA diameter at end-diastole, 2C (cm) | 3.6 | 0.4 | 3.2 | 0.3 | <0.001 | |
| MA diameter at end-systole, 2C (cm) | 3.7 | 0.4 | 3.3 | 0.4 | <0.001 | |
| MV tenting length, 3C (cm) | 0.7 | 0.2 | 0.6 | 0.1 | <0.001 | |
| MV tenting area, 3C (cm^2^) | 1.4 | 0.4 | 1.2 | 0.3 | <0.001 | |
| AML length, 3C (cm) | 2.1 | 0.2 | 2 | 0.2 | <0.001 | |
| AML (α) angle, 3C (cm) | 37 | 11 | 37 | 9 | 0.445 | |
| PML (β) angle, 3C (°) | 50 | 13 | 54 | 14 | <0.001 | |
| MA/AML ratio, 3C | 1.4 | 0.2 | 1.3 | 0.2 | <0.001 | |
| Tethering angle (β/α) ratio | 1.5 | 0.6 | 1.6 | 0.6 | 0.027 | |
| **Tricuspid valve annulus** |  |  |  |  |  |  |
| TA diameter at end-diastole, 4C (cm) | 3.2 | 0.5 | 2.9 | 0.4 | <0.001 | |
| TA diameter at end-systole, 4C (cm) | 3.2 | 0.5 | 2.8 | 0.4 | <0.001 | |
| TV tenting length, 4C (cm) | 0.4 | 0.1 | 0.3 | 0.1 | <0.001 | |
| TV tenting area, 4C (cm^2^) | 0.7 | 0.2 | 0.5 | 0.2 | <0.001 | |
| TA/MA diameter ratio at end-diastole, 4C | 1.02 | 0.18 | 0.98 | 0.15 | 0.009 | |

All values are reported in mean ± standard deviation (SD). All measurements are reported in cm (areas in cm^2^), except for ratios and angles. 2C, two-chamber; 3C, three-chamber; 4C, four-chamber; AML, anterior mitral leaflet; BSA, body surface area; MA, mitral annulus; MV, mitral valve; PML, posterior mitral leaflet; TA, tricuspid annulus; TV, tricuspid valve.

**Table S2. Normal reference ranges of mitral and tricuspid annulus measurements indexed to BSA stratified by gender.**

| **Parameter** | **Male** | | **Female** | | | **p-value** |
| --- | --- | --- | --- | --- | --- | --- |
|  | **(n=328)** | | **(n=393)** | | |  |
|  | **Mean** | **SD** | **Mean** | **SD** | |  |
| **Mitral valve annulus** |  |  |  |  |  |  |
| MA diameter at end-diastole, 4C (cm/m^2^) | 1.6 | 0.2 | 1.8 | 0.2 | <0.001 | |
| MA diameter at end-systole, 4C (cm/m^2^) | 1.7 | 0.3 | 1.8 | 0.3 | <0.001 | |
| MA diameter at end-diastole, 3C (cm/m^2^) | 1.5 | 0.2 | 1.6 | 0.2 | <0.001 | |
| MA diameter at end-systole, 3C (cm/m^2^) | 1.7 | 0.2 | 1.8 | 0.2 | <0.001 | |
| MA diameter at end-diastole, 2C (cm/m^2^) | 1.8 | 0.2 | 1.9 | 0.2 | <0.001 | |
| MA diameter at end-systole, 2C (cm/m^2^) | 1.9 | 0.2 | 1.9 | 0.2 | <0.001 | |
| MV tenting length, 3C (cm/m^2^) | 0.3 | 0.1 | 0.4 | 0.1 | 0.058 | |
| MV tenting area, 3C (cm^2^/m^2^) | 0.7 | 0.2 | 0.7 | 0.2 | 0.105 | |
| AML length, 3C (cm/m^2^) | 1.1 | 0.1 | 1.2 | 0.1 | <0.001 | |
| AML (α) angle, 3C (°/m^2^) | 19 | 6 | 22 | 6 | <0.001 | |
| PML (β) angle, 3C (°/m^2^) | 26 | 7 | 33 | 9 | <0.001 | |
| MA/AML ratio, 3C | 1.4 | 0.2 | 1.3 | 0.2 | <0.001 | |
| Tethering angle (β/α) ratio | 1.5 | 0.6 | 1.6 | 0.6 | 0.027 | |
| **Tricuspid valve annulus** |  |  |  |  |  |  |
| TA diameter at end-diastole, 4C (cm/m^2^) | 1.6 | 0.3 | 1.7 | 0.2 | <0.001 | |
| TA diameter at end-systole, 4C (cm/m^2^) | 1.7 | 0.3 | 1.7 | 0.3 | 0.395 | |
| TV tenting length, 4C (cm/m^2^) | 0.2 | 0.1 | 0.2 | 0.1 | <0.001 | |
| TV tenting area, 4C (cm^2^/m^2^) | 0.3 | 0.1 | 0.3 | 0.1 | <0.001 | |
| TA/MA diameter ratio at end-diastole, 4C | 1.02 | 0.18 | 0.98 | 0.15 | 0.009 | |

All values are reported in mean ± standard deviation (SD). All measurements are indexed to body surface area and reported in cm/m^2^ (areas in cm^2^/m^2^), except for ratios and angles. 2C, two-chamber; 3C, three-chamber; 4C, four-chamber; AML, anterior mitral leaflet; BSA, body surface area; MA, mitral annulus; MV, mitral valve; PML, posterior mitral leaflet; TA, tricuspid annulus; TV, tricuspid valve.

**Table S3. Normal reference ranges of mitral and tricuspid annulus measurements indexed to height stratified by gender.**

| **Parameter** | **Male** | | **Female** | | | **p-value** |
| --- | --- | --- | --- | --- | --- | --- |
|  | **(n=328)** | | **(n=393)** | | |  |
|  | **Mean** | **SD** | **Mean** | **SD** | |  |
| **Mitral valve annulus** |  |  |  |  |  |  |
| MA diameter at end-diastole, 4C (cm/m) | 1.8 | 0.2 | 1.8 | 0.2 | 0.545 | |
| MA diameter at end-systole, 4C (cm/m) | 1.9 | 0.3 | 1.9 | 0.2 | 0.472 | |
| MA diameter at end-diastole, 3C (cm/m) | 1.6 | 0.2 | 1.6 | 0.2 | 0.031 | |
| MA diameter at end-systole, 3C (cm/m) | 1.9 | 0.2 | 1.8 | 0.2 | 0.163 | |
| MA diameter at end-diastole, 2C (cm/m) | 2 | 0.2 | 1.9 | 0.2 | <0.001 | |
| MA diameter at end-systole, 2C (cm/m) | 2.1 | 0.2 | 2 | 0.2 | <0.001 | |
| MV tenting length, 3C (cm/m) | 0.4 | 0.1 | 0.4 | 0.1 | 0.026 | |
| MV tenting area, 3C (cm^2^/m) | 0.8 | 0.2 | 0.7 | 0.2 | <0.001 | |
| AML length, 3C (cm/m) | 1.2 | 0.1 | 1.2 | 0.1 | <0.001 | |
| AML (α) angle, 3C (°/m) | 21 | 6 | 22 | 6 | 0.004 | |
| PML (β) angle, 3C (°/m) | 28 | 8 | 33 | 8 | <0.001 | |
| MA/AML ratio, 3C | 1.4 | 0.2 | 1.3 | 0.2 | <0.001 | |
| Tethering angle (β/α) ratio | 1.5 | 0.6 | 1.6 | 0.6 | 0.027 | |
| **Tricuspid valve annulus** |  |  |  |  |  |  |
| TA diameter at end-diastole, 4C (cm/m) | 1.8 | 0.3 | 1.7 | 0.2 | <0.001 | |
| TA diameter at end-systole, 4C (cm/m) | 1.8 | 0.3 | 1.7 | 0.3 | <0.001 | |
| TV tenting length, 4C (cm/m) | 0.2 | 0.1 | 0.2 | 0.1 | <0.001 | |
| TV tenting area, 4C (cm^2^/m) | 0.4 | 0.1 | 0.3 | 0.1 | <0.001 | |
| TA/MA diameter ratio at end-diastole, 4C | 1.02 | 0.18 | 0.98 | 0.15 | 0.009 | |

All values are reported in mean ± standard deviation (SD). All measurements are reported in cm/m (areas in cm^2^/m), except for ratios and angles. 2C, two-chamber; 3C, three-chamber; 4C, four-chamber; AML, anterior mitral leaflet; BSA, body surface area; MA, mitral annulus; MV, mitral valve; PML, posterior mitral leaflet; TA, tricuspid annulus; TV, tricuspid valve.

**Table S4. Normal absolute reference ranges of mitral and tricuspid annulus measurements stratified by age group in Caucasian men.**

| **Age** | **45-54** | | **55-64** | | **65-74** | | **p-value** | **p-value for trend** |
| --- | --- | --- | --- | --- | --- | --- | --- | --- |
|  | **(n=103)** | | **(n=142)** | | **(n=83)** | |  |  |
| **Parameter** | **Mean** | **SD** | **Mean** | **SD** | **Mean** | **SD** |  |  |
| **Mitral valve annulus** |  |  |  |  |  |  |  |  |
| MA diameter at end-diastole, 4C (cm) | 3.2 | 0.4 | 3.2 | 0.5 | 3.2 | 0.4 | 0.801 | 0.672 |
| MA diameter at end-systole, 4C (cm) | 3.3 | 0.5 | 3.3 | 0.5 | 3.3 | 0.5 | 0.829 | 0.791 |
| MA diameter at end-diastole, 3C (cm) | 2.9 | 0.4 | 2.8 | 0.4 | 2.9 | 0.4 | 0.083 | 0.323 |
| MA diameter at end-systole, 3C (cm) | 3.4 | 0.4 | 3.3 | 0.4 | 3.4 | 0.4 | 0.179 | 0.807 |
| MA diameter at end-diastole, 2C (cm) | 3.6 | 0.4 | 3.6 | 0.3 | 3.6 | 0.4 | 0.621 | 0.688 |
| MA diameter at end-systole, 2C (cm) | 3.6 | 0.4 | 3.7 | 0.4 | 3.7 | 0.4 | 0.232 | 0.315 |
| MV tenting length, 3C (cm) | 0.7 | 0.1 | 0.6 | 0.2 | 0.7 | 0.2 | 0.191 | 0.274 |
| MV tenting area, 3C (cm^2^) | 1.5 | 0.3 | 1.4 | 0.4 | 1.4 | 0.4 | **0.003** | **0.009** |
| AML length, 3C (cm) | 2.1 | 0.2 | 2.1 | 0.2 | 2.1 | 0.2 | 0.386 | 0.174 |
| AML (α) angle, 3C (°) | 38 | 10 | 37 | 12 | 38 | 12 | 0.971 | 0.832 |
| PML (β) angle, 3C (°) | 54 | 12 | 50 | 14 | 46 | 11 | **<0.001** | **<0.001** |
| MA/AML ratio, 3C | 1.4 | 0.2 | 1.3 | 0.2 | 1.4 | 0.2 | 0.151 | 0.926 |
| Tethering angle (β/α) ratio | 1.6 | 0.7 | 1.5 | 0.6 | 1.3 | 0.6 | 0.066 | **0.022** |
| **Tricuspid valve annulus** |  |  |  |  |  |  |  |  |
| TA diameter at end-diastole, 4C (cm) | 3.1 | 0.5 | 3.2 | 0.5 | 3.3 | 0.5 | 0.044 | 0.081 |
| TA diameter at end-systole, 4C (cm) | 3.2 | 0.5 | 3.20 | 0.5 | 3.3 | 0.5 | 0.101 | 0.119 |
| TV tenting length, 4C (cm) | 0.4 | 0.1 | 0.4 | 0.1 | 0.3 | 0.1 | **0.014** | **0.008** |
| TV tenting area, 4C (cm^2^) | 0.7 | 0.2 | 0.7 | 0.2 | 0.6 | 0.2 | 0.801 | 0.472 |
| TA/MA diameter ratio at end-diastole, 4C | 1.0 | 0.2 | 1.0 | 0.2 | 1.1 | 0.2 | 0.042 | 0.016 |

All values are reported in mean ± standard deviation (SD). All measurements are indexed to body surface area and reported in cm/m^2^ (areas in cm^2^/m^2^), except for ratios and angles. 2C, two-chamber; 3C, three-chamber; 4C, four-chamber; AML, anterior mitral leaflet; BSA, body surface area; MA, mitral annulus; MV, mitral valve; PML, posterior mitral leaflet; TA, tricuspid annulus; TV, tricuspid valve.

**Table S5. Normal reference ranges of mitral and tricuspid annulus measurements indexed to BSA stratified by age group in Caucasian men.**

| **Age** | **45-54** | | **55-64** | | **65-74** | | **p-value** | **p-value**  **for trend** |
| --- | --- | --- | --- | --- | --- | --- | --- | --- |
|  | **(n=103)** | | **(n=142)** | | **(n=83)** | |  |  |
| **Parameter** | **Mean** | **SD** | **Mean** | **SD** | **Mean** | **SD** |  |  |
| **Mitral valve annulus** |  |  |  |  |  |  |  |  |
| MA diameter at end-diastole, 4C (cm/m^2^) | 1.6 | 0.2 | 1.6 | 0.3 | 1.7 | 0.2 | 0.375 | 0.273 |
| MA diameter at end-systole, 4C (cm/m^2^) | 1.7 | 0.2 | 1.7 | 0.3 | 1.7 | 0.3 | 0.345 | 0.233 |
| MA diameter at end-diastole, 3C (cm/m^2^) | 1.5 | 0.2 | 1.4 | 0.2 | 1.5 | 0.2 | 0.059 | 0.728 |
| MA diameter at end-systole, 3C (cm/m^2^) | 1.7 | 0.2 | 1.7 | 0.2 | 1.8 | 0.2 | **0.047** | 0.139 |
| MA diameter at end-diastole, 2C (cm/m^2^) | 1.8 | 0.2 | 1.8 | 0.2 | 1.9 | 0.2 | 0.063 | **0.035** |
| MA diameter at end-systole, 2C (cm/m^2^) | 1.8 | 0.2 | 1.9 | 0.2 | 2 | 0.2 | **0.003** | **0.002** |
| MV tenting length, 3C (cm/m^2^) | 0.3 | 0.1 | 0.3 | 0.1 | 0.3 | 0.1 | 0.242 | 0.851 |
| MV tenting area, 3C (cm^2^/m^2^) | 0.8 | 0.2 | 0.7 | 0.2 | 0.7 | 0.2 | **0.012** | **0.068** |
| AML length, 3C (cm/m^2^) | 1.1 | 0.1 | 1.1 | 0.1 | 1.1 | 0.1 | 0.669 | 0.604 |
| AML (α) angle, 3C (°/m^2^) | 19 | 5 | 19 | 6 | 20 | 7 | 0.661 | 0.76 |
| PML (β) angle, 3C (°/m^2^) | 28 | 7 | 26 | 7 | 24 | 6 | **0.003** | **0.002** |
| MA/AML ratio, 3C | 1.4 | 0.2 | 1.3 | 0.2 | 1.4 | 0.2 | 0.151 | 0.926 |
| Tethering angle (β/α) ratio | 1.6 | 0.7 | 1.5 | 0.6 | 1.3 | 0.6 | 0.066 | **0.022** |
| **Tricuspid valve annulus** |  |  |  |  |  |  |  |  |
| TA diameter at end-diastole, 4C (cm/m^2^) | 1.6 | 0.2 | 1.6 | 0.3 | 1.7 | 0.3 | **<0.001** | **<0.001** |
| TA diameter at end-systole, 4C (cm/m^2^) | 1.6 | 0.3 | 1.6 | 0.3 | 1.7 | 0.3 | **0.005** | **0.007** |
| TV tenting length, 4C (cm/m^2^) | 0.2 | 0.1 | 0.2 | 0.1 | 0.2 | 0.1 | 0.072 | 0.052 |
| TV tenting area, 4C (cm^2^/m^2^) | 0.3 | 0.1 | 0.3 | 0.1 | 0.3 | 0.1 | 0.989 | 0.941 |
| TA/MA diameter ratio at end-diastole, 4C | 1.0 | 0.2 | 1.0 | 0.2 | 1.1 | 0.2 | 0.042 | 0.016 |

All values are reported in mean ± standard deviation (SD). All measurements are indexed to body surface area and reported in cm/m^2^ (areas in cm^2^/m^2^), except for ratios and angles. 2C, two-chamber; 3C, three-chamber; 4C, four-chamber; AML, anterior mitral leaflet; BSA, body surface area; MA, mitral annulus; MV, mitral valve; PML, posterior mitral leaflet; TA, tricuspid annulus; TV, tricuspid valve.

**Table S6. Normal absolute reference ranges of mitral and tricuspid annulus measurements stratified by age group in Caucasian women.**

| **Age** | **45-54** | | **55-64** | | **65-74** | | **p-value** | **p-value for trend** |
| --- | --- | --- | --- | --- | --- | --- | --- | --- |
|  | **(n=121)** | | **(n=160)** | | **(n=112)** | |  |  |
| **Parameter** | **Mean** | **SD** | **Mean** | **SD** | **Mean** | **SD** |  |  |
| **Mitral valve annulus** |  |  |  |  |  |  |  |  |
| MA diameter at end-diastole, 4C (cm) | 3 | 0.3 | 2.90 | 0.4 | 2.9 | 0.4 | 0.074 | **0.033** |
| MA diameter at end-systole, 4C (cm) | 3.1 | 0.3 | 3.1 | 0.4 | 3 | 0.4 | 0.373 | 0.15 |
| MA diameter at end-diastole, 3C (cm) | 2.6 | 0.4 | 2.6 | 0.4 | 2.6 | 0.3 | 0.590 | 0.392 |
| MA diameter at end-systole, 3C (cm) | 3 | 0.4 | 3.1 | 0.4 | 3 | 0.4 | 0.745 | 0.669 |
| MA diameter at end-diastole, 2C (cm) | 3.2 | 0.3 | 3.2 | 0.3 | 3.2 | 0.3 | 0.178 | **0.04** |
| MA diameter at end-systole, 2C (cm) | 3.3 | 0.3 | 3.3 | 0.4 | 3.2 | 0.4 | 0.582 | 0.323 |
| MV tenting length, 3C (cm) | 0.6 | 0.2 | 0.6 | 0.1 | 0.6 | 0.1 | 0.561 | 0.505 |
| MV tenting area, 3C (cm^2^) | 1.2 | 0.3 | 1.2 | 0.3 | 1.2 | 0.3 | 0.252 | 0.396 |
| AML length, 3C (cm) | 2 | 0.2 | 2 | 0.2 | 2 | 0.2 | 0.730 | 0.383 |
| AML (α) angle, 3C (°) | 36 | 9 | 38 | 10 | 37 | 9 | 0.161 | 0.385 |
| PML (β) angle, 3C (°) | 56 | 12 | 55 | 15 | 52 | 14 | **0.043** | **0.007** |
| MA/AML ratio, 3C | 1.3 | 0.2 | 1.3 | 0.2 | 1.3 | 0.2 | 0.331 | 0.156 |
| Tethering angle (β/α) ratio | 1.7 | 0.6 | 1.6 | 0.6 | 1.5 | 0.6 | 0.064 | **0.013** |
| **Tricuspid valve annulus** |  |  |  |  |  |  |  |  |
| TA diameter at end-diastole, 4C (cm) | 2.8 | 0.4 | 2.8 | 0.4 | 3 | 0.4 | **0.002** | **0.004** |
| TA diameter at end-systole, 4C (cm) | 2.8 | 0.4 | 2.80 | 0.4 | 2.9 | 0.5 | 0.442 | 0.275 |
| TV tenting length, 4C (cm) | 0.3 | 0.1 | 0.3 | 0.1 | 0.3 | 0.1 | 0.661 | 0.203 |
| TV tenting area, 4C (cm^2^) | 0.5 | 0.1 | 0.5 | 0.2 | 0.4 | 0.2 | 0.379 | 0.059 |
| TA/MA diameter ratio at end-diastole, 4C | 0.9 | 0.1 | 1.0 | 0.2 | 1.0 | 0.2 | <0.001 | <0.001 |

All values are reported in mean ± standard deviation (SD). All measurements are reported in cm (areas in cm^2^), except for ratios and angles. 2C, two-chamber; 3C, three-chamber; 4C, four-chamber; AML, anterior mitral leaflet; BSA, body surface area; MA, mitral annulus; MV, mitral valve; PML, posterior mitral leaflet; TA, tricuspid annulus; TV, tricuspid valve.

**Table S7. Normal reference ranges of mitral and tricuspid annulus measurements indexed to BSA stratified by age group in Caucasian women.**

| **Age** | **45-54** | | **55-64** | | **65-74** | | **p-value** | **p-value**  **for trend** |
| --- | --- | --- | --- | --- | --- | --- | --- | --- |
|  | **(n=121)** | | **(n=160)** | | **(n=112)** | |  |  |
| **Parameters** | **Mean** | **SD** | **Mean** | **SD** | **Mean** | **SD** |  |  |
| **Mitral valve annulus** |  |  |  |  |  |  |  |  |
| MA diameter at end-diastole, 4C (cm/m^2^) | 1.8 | 0.2 | 1.7 | 0.2 | 1.8 | 0.3 | 0.355 | 0.615 |
| MA diameter at end-systole, 4C (cm/m^2^) | 1.8 | 0.2 | 1.8 | 0.2 | 1.8 | 0.3 | 0.973 | 0.956 |
| MA diameter at end-diastole, 3C (cm/m^2^) | 1.5 | 0.2 | 1.6 | 0.2 | 1.6 | 0.2 | 0.123 | **0.034** |
| MA diameter at end-systole, 3C (cm/m^2^) | 1.8 | 0.2 | 1.8 | 0.2 | 1.8 | 0.2 | 0.216 | 0.083 |
| MA diameter at end-diastole, 2C (cm/m^2^) | 1.9 | 0.2 | 1.9 | 0.2 | 1.9 | 0.2 | 0.922 | 0.727 |
| MA diameter at end-systole, 2C (cm/m^2^) | 1.9 | 0.2 | 2 | 2 | 2 | 0.3 | 0.912 | 0.817 |
| MV tenting length, 3C (cm/m^2^) | 0.3 | 0.1 | 0.4 | 0.1 | 0.4 | 0.1 | 0.256 | 0.261 |
| MV tenting area, 3C (cm^2^/m^2^) | 0.7 | 0.2 | 0.7 | 0.2 | 0.7 | 0.2 | 0.329 | 0.741 |
| AML length, 3C (cm/m^2^) | 1.2 | 0.1 | 1.2 | 0.1 | 1.2 | 0.1 | 0.733 | 0.468 |
| AML (α) angle, 3C (°/m^2^) | 21 | 6 | 23 | 6 | 22 | 5 | 0.124 | 0.198 |
| PML (β) angle, 3C (°/m^2^) | 33 | 8 | 33 | 9 | 31 | 9 | 0.159 | **0.04** |
| MA/AML ratio, 3C | 1.3 | 0.2 | 1.3 | 0.2 | 1.3 | 0.2 | 0.331 | 0.156 |
| Tethering angle (β/α) ratio | 1.7 | 0.6 | 1.6 | 0.6 | 1.5 | 0.6 | 0.064 | **0.013** |
| **Tricuspid valve annulus** |  |  |  |  |  |  |  |  |
| TA diameter at end-diastole, 4C (cm/m^2^) | 1.7 | 0.2 | 1.7 | 0.2 | 1.8 | 0.3 | **<0.001** | **<0.001** |
| TA diameter at end-systole, 4C (cm/m^2^) | 1.7 | 0.3 | 1.7 | 0.3 | 1.7 | 0.3 | 0.161 | 0.059 |
| TV tenting length, 4C (cm/m^2^) | 0.2 | 0.1 | 0.2 | 0.1 | 0.2 | 0.1 | 0.883 | 0.4 |
| TV tenting area, 4C (cm^2^/m^2^) | 0.3 | 0.1 | 0.3 | 0.1 | 0.3 | 0.1 | 0.649 | 0.157 |
| TA/MA diameter ratio at end-diastole, 4C | 0.9 | 0.1 | 1.0 | 0.2 | 1.0 | 0.2 | <0.001 | <0.001 |

All values are reported in mean ± standard deviation (SD). All measurements are indexed to body surface area and reported in cm/m^2^ (areas in cm^2^/m^2^), except for ratios and angles. 2C, two-chamber; 3C, three-chamber; 4C, four-chamber; AML, anterior mitral leaflet; BSA, body surface area; MA, mitral annulus; MV, mitral valve; PML, posterior mitral leaflet; TA, tricuspid annulus; TV, tricuspid valve.

**Table S8**. **Normal reference ranges of mitral and tricuspid annulus measurements indexed to height stratified by age group in Caucasian men.**

| **Age** | **45-54** | | **55-64** | | **65-74** | | **p-value** | **p-value for trend** |
| --- | --- | --- | --- | --- | --- | --- | --- | --- |
|  | **(n=103)** | | **(n=142)** | | **(n=83)** | |  |  |
| **Parameters** | **Mean** | **SD** | **Mean** | **SD** | **Mean** | **SD** |  |  |
| **Mitral valve annulus** |  |  |  |  |  |  |  |  |
| MA diameter at end-diastole, 4C (cm/m) | 1.8 | 0.2 | 1.8 | 0.3 | 1.8 | 0.2 | 0.822 | 0.85 |
| MA diameter at end-systole, 4C (cm/m) | 1.9 | 0.2 | 1.8 | 0.3 | 1.9 | 0.3 | 0.770 | 0.672 |
| MA diameter at end-diastole, 3C (cm/m) | 1.6 | 0.2 | 1.6 | 0.2 | 1.6 | 0.2 | 0.121 | 0.622 |
| MA diameter at end-systole, 3C (cm/m) | 1.9 | 0.2 | 1.8 | 0.2 | 1.9 | 0.2 | 0.173 | 0.604 |
| MA diameter at end-diastole, 2C (cm/m) | 2 | 0.2 | 2 | 0.2 | 2 | 0.2 | 0.471 | 0.375 |
| MA diameter at end-systole, 2C (cm/m) | 2 | 0.2 | 2.1 | 0.2 | 2.1 | 0.2 | **0.041** | **0.028** |
| MV tenting length, 3C (cm/m) | 0.4 | 0.1 | 0.4 | 0.1 | 0.4 | 0.1 | 0.247 | 0.424 |
| MV tenting area, 3C (cm^2^/m) | 0.9 | 0.2 | 0.8 | 0.2 | 0.8 | 0.2 | **0.006** | **0.02** |
| AML length, 3C (cm/m) | 1.2 | 0.1 | 1.2 | 0.1 | 1.2 | 0.1 | 0.891 | 0.501 |
| AML (α) angle, 3C (°/m) | 21 | 6 | 21 | 7 | 21 | 7 | 0.921 | 0.99 |
| PML (β) angle, 3C (°/m) | 30 | 7 | 28 | 8 | 26 | 6 | **<0.001** | **<0.001** |
| MA/AML ratio, 3C | 1.4 | 0.2 | 1.3 | 0.2 | 1.4 | 0.2 | 0.151 | 0.926 |
| Tethering angle (β/α) ratio | 1.6 | 0.7 | 1.5 | 0.6 | 1.3 | 0.6 | 0.066 | **0.022** |
| **Tricuspid valve annulus** |  |  |  |  |  |  |  |  |
| TA diameter at end-diastole, 4C (cm/m) | 1.8 | 0.3 | 1.80 | 0.3 | 1.9 | 0.3 | **0.010** | **0.008** |
| TA diameter at end-systole, 4C (cm/m) | 1.8 | 0.3 | 1.80 | 0.3 | 1.9 | 0.3 | **0.032** | **0.029** |
| TV tenting length, 4C (cm/m) | 0.2 | 0.1 | 0.2 | 0.1 | 0.2 | 0.1 | **0.025** | **0.021** |
| TV tenting area, 4C (cm^2^/m) | 0.4 | 0.1 | 0.4 | 0.1 | 0.4 | 0.1 | 0.903 | 0.69 |
| TA/MA diameter ratio at end-diastole, 4C | 1.0 | 0.2 | 1.0 | 0.2 | 1.1 | 0.2 | 0.042 | 0.016 |

All values are reported in mean ± standard deviation (SD). All measurements are indexed to height and reported in cm/m (areas in cm^2^/m), except for ratios and angles. 2C, two-chamber; 3C, three-chamber; 4C, four-chamber; AML, anterior mitral leaflet; BSA, body surface area; MA, mitral annulus; MV, mitral valve; PML, posterior mitral leaflet; TA, tricuspid annulus; TV, tricuspid valve.

**Table S9. Normal reference ranges of mitral and tricuspid annulus measurements indexed to height stratified by age group in Caucasian women.**

| **Age** | **45-54** | | **55-64** | | **65-74** | | **p-value** | **p-value for**  **trend** |
| --- | --- | --- | --- | --- | --- | --- | --- | --- |
|  | **(n=121)** | | **(n=160)** | | **(n=112)** | |  |  |
| **Parameters** | **Mean** | **SD** | **Mean** | **SD** | **Mean** | **SD** |  |  |
| **Mitral valve annulus** |  |  |  |  |  |  |  |  |
| MA diameter at end-diastole, 4C (cm/m) | 1.8 | 0.2 | 1.8 | 0.2 | 1.8 | 0.2 | 0.221 | 0.498 |
| MA diameter at end-systole, 4C (cm/m) | 1.9 | 0.2 | 1.9 | 0.2 | 1.9 | 0.3 | 0.937 | 0.805 |
| MA diameter at end-diastole, 3C (cm/m) | 1.6 | 0.2 | 1.6 | 0.2 | 1.6 | 0.2 | 0.108 | **0.042** |
| MA diameter at end-systole, 3C (cm/m) | 1.8 | 0.2 | 1.9 | 0.2 | 1.9 | 0.2 | 0.204 | 0.12 |
| MA diameter at end-diastole, 2C (cm/m) | 1.9 | 0.2 | 1.9 | 0.2 | 1.9 | 0.2 | 0.841 | 0.474 |
| MA diameter at end-systole, 2C (cm/m) | 2 | 0.2 | 2 | 0.2 | 2 | 0.3 | 0.936 | 0.804 |
| MV tenting length, 3C (cm/m) | 0.3 | 0.1 | 0.4 | 0.1 | 0.4 | 0.1 | 0.295 | 0.239 |
| MV tenting area, 3C (cm^2^/m) | 0.7 | 0.2 | 0.7 | 0.2 | 0.7 | 0.2 | 0.345 | 0.696 |
| AML length, 3C (cm/m) | 1.2 | 0.1 | 1.2 | 0.1 | 1.2 | 0.1 | 0.680 | 0.421 |
| AML (α) angle, 3C (°/m) | 22 | 6 | 23 | 6 | 22 | 5 | 0.112 | 0.181 |
| PML (β) angle, 3C (°/m) | 34 | 7 | 34 | 9.1 | 32 | 9 | 0.146 | **0.037** |
| MA/AML ratio, 3C | 1.3 | 0.2 | 1.3 | 0.2 | 1.3 | 0.2 | 0.331 | 0.156 |
| Tethering angle (β/α) ratio | 1.7 | 0.6 | 1.6 | 0.6 | 1.5 | 0.6 | 0.064 | **0.013** |
| **Tricuspid valve annulus** |  |  |  |  |  |  |  |  |
| TA diameter at end-diastole, 4C (cm/m) | 1.7 | 0.2 | 1.7 | 0.2 | 1.8 | 0.3 | **<0.001** | **<0.001** |
| TA diameter at end-systole, 4C (cm/m) | 1.7 | 0.2 | 1.7 | 0.3 | 1.7 | 0.3 | 0.122 | 0.056 |
| TV tenting length, 4C (cm/m) | 0.2 | 0.1 | 0.2 | 0.1 | 0.2 | 0.1 | 0.875 | 0.374 |
| TV tenting area, 4C (cm^2^/m) | 0.3 | 0.1 | 0.3 | 0.1 | 0.3 | 0.1 | 0.592 | 0.133 |
| TA/MA diameter ratio at end-diastole, 4C | 0.9 | 0.1 | 1.0 | 0.2 | 1.0 | 0.2 | <0.001 | <0.001 |

All values are reported in mean ± standard deviation (SD). All measurements are indexed to height and reported in cm/m (areas in cm^2^/m), except for ratios and angles. 2C, two-chamber; 3C, three-chamber; 4C, four-chamber; AML, anterior mitral leaflet; BSA, body surface area; MA, mitral annulus; MV, mitral valve; PML, posterior mitral leaflet; TA, tricuspid annulus; TV, tricuspid valve.

**Table S10. Normal reference ranges of mitral and tricuspid valve annulus measurements indexed to height in Caucasian men.**

| **Parameters** | **Abnormal low** |  | **Normal range** |  | **Abnormal high** |
| --- | --- | --- | --- | --- | --- |
| **Mitral valve annulus** |  |  |  |  |  |
| MA diameter at end-diastole, 4C (cm/m) | <1.3 | **Borderline zone** | 1.4 - 2.2 | **Borderline zone** | >2.3 |
| MA diameter at end-systole, 4C (cm/m) | <1.3 |  | 1.4 - 2.4 |  | >2.5 |
| MA diameter at end-diastole, 3C (cm/m) | <1.1 |  | 1.2 - 2 |  | >2.1 |
| MA diameter at end-systole, 3C (cm/m) | <1.4 |  | 1.5 - 2.3 |  | >2.4 |
| MA diameter at end-diastole, 2C (cm/m) | <1.6 |  | 1.6 - 2.4 |  | >2.5 |
| MA diameter at end-systole, 2C (cm/m) | <1.6 |  | 1.7 - 2.4 |  | >2.6 |
| MV tenting length, 3C (cm/m) | <0.2 |  | 0.2 - 0.6 |  | >0.6 |
| MV tenting area, 3C (cm^2^/m) | <0.4 |  | 0.5 - 1.2 |  | >1.2 |
| AML length, 3C (cm/m) | <1 |  | 1 - 1.4 |  | >1.4 |
| AML (α) angle, 3C (°/m) | <7 |  | 10 - 32 |  | >35 |
| PML (β) angle, 3C (°/m) | <12 |  | 16 – 39 |  | >45 |
| MA/AML ratio, 3C | <0.9 |  | 1 - 1.7 |  | >1.8 |
| Tethering angle (β/α) ratio | <0.2 |  | 0.3 - 2.5 |  | >2.9 |
| **Tricuspid valve annulus** |  |  |  |  |  |
| TA diameter at end-diastole, 4C (cm/m) | <1.2 |  | 1.3 - 2.3 |  | >2.5 |
| TA diameter at end-systole, 4C (cm/m) | <1.2 |  | 1.3 - 2.3 |  | >2.5 |
| TV tenting length, 4C (cm/m) | <0 |  | 0.1 - 0.3 |  | >0.4 |
| TV tenting area, 4C (cm^2^/m) | <0.1 |  | 0.1 - 0.6 |  | >0.6 |
| TA/MA diameter ratio at end-diastole, 4C | <0.7 |  | 0.7 – 1.3 |  | >1.5 |

Abnormal low and high refer to the lower and upper reference limits, respectively. They are defined as measurements which lie outside the 95% prediction interval at all age groups. Borderline zone values should be looked up in the age-specific tables. The borderline zone was defined as the upper and lower ranges where the measured value lay outside the 95% prediction interval for at least one age group. All measurements are indexed to height and reported in cm/m (areas in cm^2^/m), except for ratios and angles. 2C, two-chamber; 3C, three-chamber; 4C, four-chamber; AML, anterior mitral leaflet; BSA, body surface area; MA, mitral annulus; MV, mitral valve; PML, posterior mitral leaflet; TA, tricuspid annulus; TV, tricuspid valve.

**Table S11. Age-specific normal reference ranges of mitral and tricuspid valve annulus measurements indexed to height in Caucasian men.**

| **Parameters** |  | **45-54** |  |  | **55-64** |  |  | **65-74** |  |
| --- | --- | --- | --- | --- | --- | --- | --- | --- | --- |
| **Mitral valve annulus** | **lower** | **mean** | **upper** | **lower** | **mean** | **upper** | **lower** | **mean** | **upper** |
| MA diameter at end-diastole, 4C (cm) | 1.4 | 1.8 | 2.2 | 1.3 | 1.8 | 2.3 | 1.3 | 1.8 | 2.3 |
| MA diameter at end-systole, 4C (cm) | 1.4 | 1.9 | 2.4 | 1.3 | 1.8 | 2.4 | 1.3 | 1.9 | 2.5 |
| MA diameter at end-diastole, 3C (cm) | 1.2 | 1.6 | 2 | 1.1 | 1.6 | 2 | 1.1 | 1.6 | 2.1 |
| MA diameter at end-systole, 3C (cm) | 1.5 | 1.9 | 2.3 | 1.4 | 1.8 | 2.3 | 1.4 | 1.9 | 2.4 |
| MA diameter at end-diastole, 2C (cm) | 1.6 | 2 | 2.4 | 1.6 | 2 | 2.4 | 1.6 | 2 | 2.5 |
| MA diameter at end-systole, 2C (cm) | 1.7 | 2 | 0.6 | 1.6 | 2.1 | 2.5 | 1.6 | 2.1 | 2.6 |
| MV tenting length, 3C (cm) | 0.2 | 0.4 | 1.2 | 0.2 | 0.4 | 0.6 | 0.2 | 0.4 | 0.6 |
| MV tenting area, 3C (cm^2^) | 0.5 | 0.9 | 1.4 | 0.4 | 0.8 | 1.2 | 0.4 | 0.8 | 1.2 |
| AML length, 3C (cm) | 1 | 1.2 | 1.4 | 1 | 1.2 | 1.4 | 1 | 1.2 | 1.4 |
| AML (α) angle, 3C (°) | 10 | 21 | 32 | 7 | 21 | 35 | 7 | 21 | 35 |
| PML (β) angle, 3C (°) | 16 | 30 | 45 | 12 | 28 | 44 | 13 | 26 | 39 |
| MA/AML ratio, 3C | 1 | 1.4 | 1.8 | 0.9 | 1.3 | 1.7 | 1 | 1.4 | 1.8 |
| Tethering angle (β/α) ratio | 0.3 | 1.6 | 2.9 | 0.2 | 1.5 | 2.7 | 0.1 | 1.3 | 2.5 |
| **Tricuspid valve annulus** |  |  |  |  |  |  |  |  |  |
| TA diameter at end-diastole, 4C (cm) | 1.2 | 1.8 | 2.3 | 1.2 | 1.8 | 2.4 | 1.3 | 1.9 | 2.5 |
| TA diameter at end-systole, 4C (cm) | 1.2 | 1.8 | 2.3 | 1.2 | 1.8 | 2.4 | 1.3 | 1.9 | 2.5 |
| TV tenting length, 4C (cm) | 0.1 | 0.2 | 0.4 | 0 | 0.2 | 0.4 | 0.1 | 0.2 | 0.3 |
| TV tenting area, 4C (cm^2^) | 0.1 | 0.4 | 0.6 | 0.1 | 0.4 | 0.6 | 0.1 | 0.4 | 0.6 |
| TA/MA diameter ratio at end-diastole, 4C | 0.7 | 1.0 | 1.3 | 0.7 | 1.0 | 1.4 | 0.7 | 1.1 | 1.5 |

Male reference ranges detailing mean, lower reference limit and upper reference limit by age group. Reference limits are derived by the upper and lower bounds of the 95% prediction interval for each parameter at each age group. All measurements are indexed to height and reported in cm/m (areas in cm^2^/m), except for ratios and angles. 2C, two-chamber; 3C, three-chamber; 4C, four-chamber; AML, anterior mitral leaflet; BSA, body surface area; MA, mitral annulus; MV, mitral valve; PML, posterior mitral leaflet; TA, tricuspid annulus; TV, tricuspid valve.

**Table S12. Age-specific normal reference ranges of mitral and tricuspid valve annulus measurements indexed to height in Caucasian women.**

| **Parameters** | **Abnormal low** |  | **Normal range** |  | **Abnormal high** |
| --- | --- | --- | --- | --- | --- |
| **Mitral valve annulus** |  |  |  |  |  |
| MA diameter at end-diastole, 4C (cm/m) | <1.3 | **Borderline zone** | 1.5 - 2.1 | **Borderline zone** | >2.3 |
| MA diameter at end-systole, 4C (cm/m) | <1.3 |  | 1.5 - 2.3 |  | >2.4 |
| MA diameter at end-diastole, 3C (cm/m) | <1.1 |  | 1.2 - 2 |  | >2 |
| MA diameter at end-systole, 3C (cm/m) | <1.4 |  | 1.4 - 2.2 |  | >2.3 |
| MA diameter at end-diastole, 2C (cm/m) | <1.5 |  | 1.6 - 2.3 |  | >2.3 |
| MA diameter at end-systole, 2C (cm/m) | <1.5 |  | 1.6 - 2.3 |  | >2.5 |
| MV tenting length, 3C (cm/m) | <0.2 |  | 0.2 - 0.5 |  | >0.5 |
| MV tenting area, 3C (cm^2^/m) | <0.3 |  | 0.4 - 1.1 |  | >1.1 |
| AML length, 3C (cm/m) | <1 |  | 1 - 1.4 |  | >1.5 |
| AML (α) angle, 3C (°/m) | <10 |  | 12 - 32 |  | >35 |
| PML (β) angle, 3C (°/m) | <14 |  | 19 - 49 |  | >52 |
| MA/AML ratio, 3C | <0.9 |  | 0.9 - 1.6 |  | >1.7 |
| Tethering angle (β/α) ratio | <0.3 |  | 0.5 - 2.6 |  | >2.9 |
| **Tricuspid valve annulus** |  |  |  |  |  |
| TA diameter at end-diastole, 4C (cm/m) | <1.2 |  | 1.3 - 2.1 |  | >2.3 |
| TA diameter at end-systole, 4C (cm/m) | <1.2 |  | 1.2 - 2.2 |  | >2.3 |
| TV tenting length, 4C (cm/m) | <0 |  | 0.1 - 0.3 |  | >0.3 |
| TV tenting area, 4C (cm^2^/m) | <0.1 |  | 0.1 - 0.5 |  | >0.5 |
| TA/MA diameter ratio at end-diastole, 4C | <0.7 |  | 0.7 - 1.2 |  | >1.4 |

Abnormal low and high refer to the lower and upper reference limits, respectively. They are defined as measurements which lie outside the 95% prediction interval at all age groups. Borderline zone values should be looked up in the age-specific tables. The borderline zone was defined as the upper and lower ranges where the measured value lay outside the 95% prediction interval for at least one age group. All measurements are indexed to height and reported in cm/m (areas in cm^2^/m), except for ratios and angles. 2C, two-chamber; 3C, three-chamber; 4C, four-chamber; AML, anterior mitral leaflet; BSA, body surface area; MA, mitral annulus; MV, mitral valve; PML, posterior mitral leaflet; TA, tricuspid annulus; TV, tricuspid valve.

**Table S13. Age-specific normal reference ranges of mitral and tricuspid valve annulus measurements indexed to height in Caucasian women.**

| **Parameters** |  | **45-54** |  |  | **55-64** |  |  | **65-74** |  |
| --- | --- | --- | --- | --- | --- | --- | --- | --- | --- |
| **Mitral valve annulus** | **lower** | **mean** | **upper** | **lower** | **mean** | **upper** | **lower** | **mean** | **upper** |
| MA diameter at end-diastole, 4C (cm/m) | 1.5 | 1.8 | 2.1 | 1.4 | 1.8 | 2.2 | 1.3 | 1.8 | 2.3 |
| MA diameter at end-systole, 4C (cm/m) | 1.5 | 1.9 | 2.3 | 1.4 | 1.9 | 2.3 | 1.3 | 1.9 | 2.4 |
| MA diameter at end-diastole, 3C (cm/m) | 1.1 | 1.6 | 2 | 1.2 | 1.6 | 2 | 1.2 | 1.6 | 2 |
| MA diameter at end-systole, 3C (cm/m) | 1.4 | 1.8 | 2.2 | 1.4 | 1.9 | 2.3 | 1.4 | 1.9 | 2.3 |
| MA diameter at end-diastole, 2C (cm/m) | 1.6 | 1.9 | 2.3 | 1.6 | 1.9 | 2.3 | 1.5 | 1.9 | 2.3 |
| MA diameter at end-systole, 2C (cm/m) | 1.6 | 2 | 2.3 | 1.6 | 2 | 2.4 | 1.5 | 2 | 2.5 |
| MV tenting length, 3C (cm/m) | 0.2 | 0.3 | 0.5 | 0.2 | 0.4 | 0.5 | 0.2 | 0.4 | 0.5 |
| MV tenting area, 3C (cm^2^/m) | 0.3 | 0.7 | 1.1 | 0.4 | 0.7 | 1.1 | 0.3 | 0.7 | 1.1 |
| AML length, 3C (cm/m) | 1 | 1.2 | 1.4 | 1 | 1.2 | 1.5 | 1 | 1.2 | 1.4 |
| AML (α) angle, 3C (°/m) | 10 | 22 | 33 | 11 | 23 | 35 | 12 | 22 | 33 |
| PML (β) angle, 3C (°/m) | 19 | 34 | 49 | 15 | 34 | 52 | 14 | 32 | 50 |
| MA/AML ratio, 3C | 0.9 | 1.3 | 1.6 | 0.9 | 1.3 | 1.7 | 0.9 | 1.3 | 1.7 |
| Tethering angle (β/α) ratio | 0.5 | 1.7 | 2.9 | 0.3 | 1.6 | 2.8 | 0.3 | 1.5 | 2.6 |
| **Tricuspid valve annulus** |  |  |  |  |  |  |  |  |  |
| TA diameter at end-diastole, 4C (cm/m) | 1.2 | 1.7 | 2.1 | 1.3 | 1.7 | 2.2 | 1.3 | 1.8 | 2.3 |
| TA diameter at end-systole, 4C (cm/m) | 1.2 | 1.7 | 2.2 | 1.2 | 1.7 | 2.2 | 1.2 | 1.7 | 2.3 |
| TV tenting length, 4C (cm/m) | 0.1 | 0.2 | 0.3 | 0 | 0.2 | 0.3 | 0 | 0.2 | 0.3 |
| TV tenting area, 4C (cm^2^/m) | 0.1 | 0.3 | 0.5 | 0.1 | 0.3 | 0.5 | 0.1 | 0.3 | 0.5 |
| TA/MA diameter ratio at end-diastole, 4C | 0.7 | 0.9 | 1.2 | 0.7 | 1.0 | 1.3 | 0.7 | 1.0 | 1.4 |

Female reference ranges detailing mean, lower reference limit and upper reference limit by age group. Reference limits are derived by the upper and lower bounds of the 95% prediction interval for each parameter at each age group. All measurements are indexed to height and reported in cm/m (areas in cm^2^/m), except for ratios and angles. 2C, two-chamber; 3C, three-chamber; 4C, four-chamber; AML, anterior mitral leaflet; BSA, body surface area; MA, mitral annulus; MV, mitral valve; PML, posterior mitral leaflet; TA, tricuspid annulus; TV, tricuspid valve.
